# Supplementary material for: Structure-guided engineering of biased-agonism in the human niacin receptor via single amino acid substitution
Source: Nat Commun. 2024 Mar 2;15:1939. doi: 10.1038/s41467-024-46239-2 (PMC10908815; doi:10.1038/s41467-024-46239-2)

Supplementary Information

Structure-guided engineering of biased-agonism in the human niacin receptor via single amino acid substitution

Manish K. Yadav[1#], Parishmita Sarma[1#], Jagannath Maharana[1], Manisankar Ganguly[1], Sudha Mishra[1], Nashrah Zaidi[1], Annu Dalal[1], Vinay Singh[1], Sayantan Saha[1], Gargi Mahajan[1], Saloni Sharma[1], Mohamed Chami[2], Ramanuj Banerjee[1*] and Arun K. Shukla[1*]

# These authors contributed equally

[1]Department of Biological Sciences and Bioengineering, Indian Institute of Technology, Kanpur 208016, India; [2]BioEM Lab, Biozentrum, Universität Basel, Basel, Switzerland.

*Corresponding authors (ramanujb@iitk.ac.in or arshukla@iitk.ac.in)

- Supplementary Fig. 1

- Supplementary Fig. 2

- Supplementary Fig. 3

- Supplementary Fig. 4

- Supplementary Fig. 5

- Supplementary Fig. 6

- Supplementary Fig. 7

- Supplementary Fig. 8

- Supplementary Fig. 9

- Supplementary Fig. 10

- Supplementary Fig. 11

- Supplementary Fig. 12

- Supplementary Fig. 13

- Supplementary Fig. 14

- Supplementary Fig. 15

- Supplementary Fig. 16

- Supplementary Fig. 16

- Supplementary Fig. 17

- Supplementary Table 1

- Source Data file : Supplementary Fig. 3a, 3b, 3c

- Source Data file : Supplementary Fig. 4a, 4b

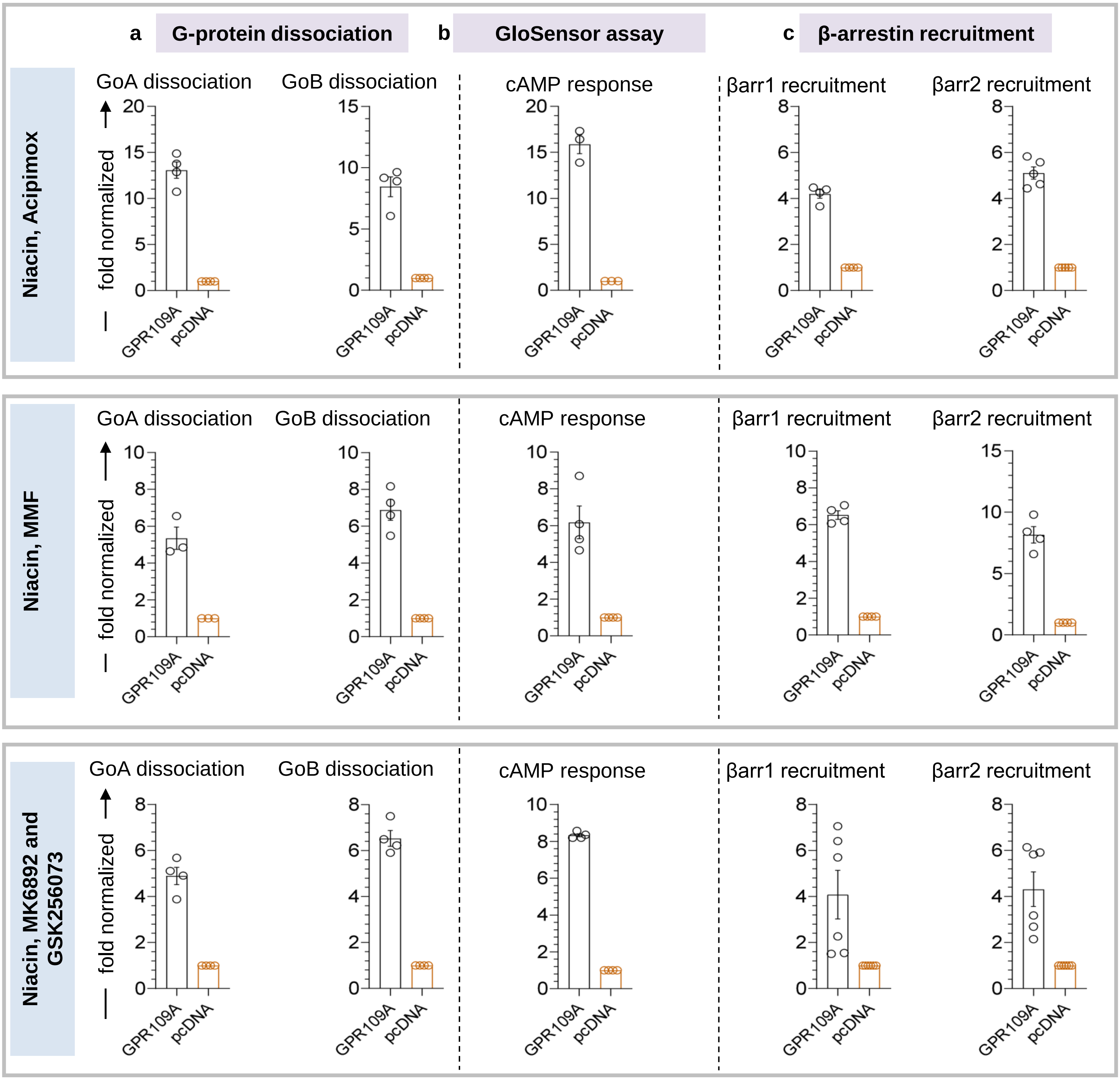

Supplementary Fig. 1. Surface expression of GPR109A in various assays. a, Surface expression of GPR109A in Go_A/ Go_B dissociation assay was measured using whole cell based surface ELISA (mean±SEM; n=3-4 independent experiments, i.e., for G_{oA} activation in response to acipimox, MK6892, and GSK256073 and G_{oB} activation in response to acipimox, MMF, MK6892, and GSK256073: n=4 and for Go_A dissociation in response to MMF: n=3; normalized as fold over pcDNA) **b,** Surface expression of GPR109A in GloSensor assay (mean±SEM; n=3-4 independent experiments, i.e., for cAMP response with MMF, MK6892, and GSK256073: n=4 and for acipimox stimulated response: n=3; normalized as fold over pcDNA) **c,** Surface expression in βarr1/2 recruitment assay (mean±SEM; n=4-6 independent experiments, for βarr1 recruitment in response to acipimox, and βarr1/2 recruitment in response to MMF: n=4 , for βarr2 recruitment in response to acipimox: n=5, for βarr1/2 recruitment in response to MK6892 and GSK256073: n=6; normalized as fold over pcDNA). Left corner of the box shows the respective ligands used in the assays. Source data is provided as source data file.

a

Ligand	Go_A dissociation		Go_B dissociation		cAMP response		β-arrestin1 recruitment		β-arrestin2 recruitment	
	EC_{50}	E_{max}	EC_{50}	E_{max}	EC_{50}	E_{max}	EC_{50}	E_{max}	EC_{50}	E_{max}
Niacin	1.34±0.52 ×10^{-7}	0.74 ±0.02	2.42± 0.91×10^{-7}	0.68± 0.03	8.07±3.35 ×10-8	71.50± 1.99	7.90±1.95 ×10^{-8}	1.96± 0.04	5.09±1.36 ×10-8	1.72± 0.03
Acipimox	3.93±2.40 ×10^{-6}	0.70± 0.08	3.54±2.44 ×10^{-6}	0.65± 0.10	1.86±0.73 ×10^{-6}	71.09± 7.40	2.29±0.76 ×10^{-6}	2.05± 0.11	1.00±0.33 ×10^{-6}	1.61± 0.05
Niacin	2.35±0.58 x10^{-7}	0.72± 0.01	2.35±0.95 x10^{-7}	0.72± 0.02	8.94±4.51 x10^{-8}	72.34± 2.39	6.13±1.44 x10^{-8}	2.15± 0.04	4.98±0.74 x10^{-8}	1.88± 0.04
MMF	7.00±1.52 x10^{-7}	0.69± 0.02	7.34±1.93 x10^{-7}	0.69± 0.02	2.59±0.79 x10^{-7}	64.39± 2.08	4.80±0.69 x10^{-7}	2.39± 0.04	3.19±0.61 x10^{-7}	1.85±0. 04
Niacin	4.22±1.14 ×10^{-7}	0.74± 0.02	3.34±1.10 ×10^{-7}	0.73± 0.02	3.48±1.22 ×10^{-8}	59.76± 4.37	1.02±0.29 ×10^{-7}	2.05± 0.05	5.48±2.45 ×10^{-8}	1.69± 0.04
MK6892	9.18±1.14 ×10^{-7}	0.50± 0.02	5.64±0.95 ×10^{-7}	0.47± 0.02	2.89±0.63 ×10^{-8}	22.69± 2.19	2.71±0.77 ×10^{-7}	2.05± 0.06	2.87±1.10 ×10^{-7}	1.69± 0.05
GSK256073	2.00±0.64 ×10^{-7}	0.55± 0.03	2.71±0.55 ×10^{-7}	0.54± 0.02	3.29±0.70 ×10^{-8}	0.54± 1.84	5.32±1.91 ×10^{-8}	2.49± 0.08	2.95±1.54 ×10^{-8}	1.88±0. 06

b

Niacin vs. MK6892

Transducer coupling	Bias factor	Mean	SEM
G_{oA} dissociation vs. βarr1 recruitment	β ± Δβ	0.343	0.193
G_{oB} dissociation vs. βarr1 recruitment	β ± Δβ	0.683	0.252
cAMP response vs. βarr1 recruitment	β ± Δβ	1.08	0.21

Niacin vs. MK6892

Transducer coupling	Bias factor	Mean	SEM
G_{oA} dissociation vs. βarr2 recruitment	β ± Δβ	0.633	0.244
G_{oB} dissociation vs. βarr2 recruitment	β ± Δβ	0.965	0.292
cAMP response vs. βarr2 recruitment	β ± Δβ	1.37	0.257

c

Niacin vs. MMF

Transducer coupling	Bias factor	Mean	SEM
G_{oA} dissociation vs. βarr1 recruitment	β ± Δβ	0.503	0.169
G_{oB} dissociation vs. βarr1 recruitment	β ± Δβ	0.545	0.243
cAMP response vs. βarr1 recruitment	β ± Δβ	0.597	0.245

Niacin vs. MMF

Transducer coupling	Bias factor	Mean	SEM
G_{oA} dissociation vs. βarr2 recruitment	β ± Δβ	0.466	0.162
G_{oB} dissociation vs. βarr2 recruitment	β ± Δβ	0.508	0.238
cAMP response vs. βarr2 recruitment	β ± Δβ	0.560	0.243

Supplementary Fig. 2. a, EC_{50} and E_{max} values for each assay in response to different ligands is shown in the table. **b, c,** Bias factor was calculated using the software https://biasedcalculator.shinyapps.io/calc/ (Detailed formula is provided in methods section). The compiled data of G-protein activation and βarr1 recruitment assays (mean ± SEM, n=3-5 independent experiments; In response to MK6892, n=4 for G_{oA}, G_{oB} dissociation, cAMP response and n=6 for βarr1/2 recruitment; in response to MMF, n=3 for G_{oA} dissociation, n=4 for G_{oB} dissociation, cAMP response and βarr1 recruitment; and n=5 for βarr2 recruitment) was used for the calculation. During bias factor calculation niacin stimulated response was considered as reference and observed G-protein biased with MK6892 and MMF. Source data is provided as source data file.

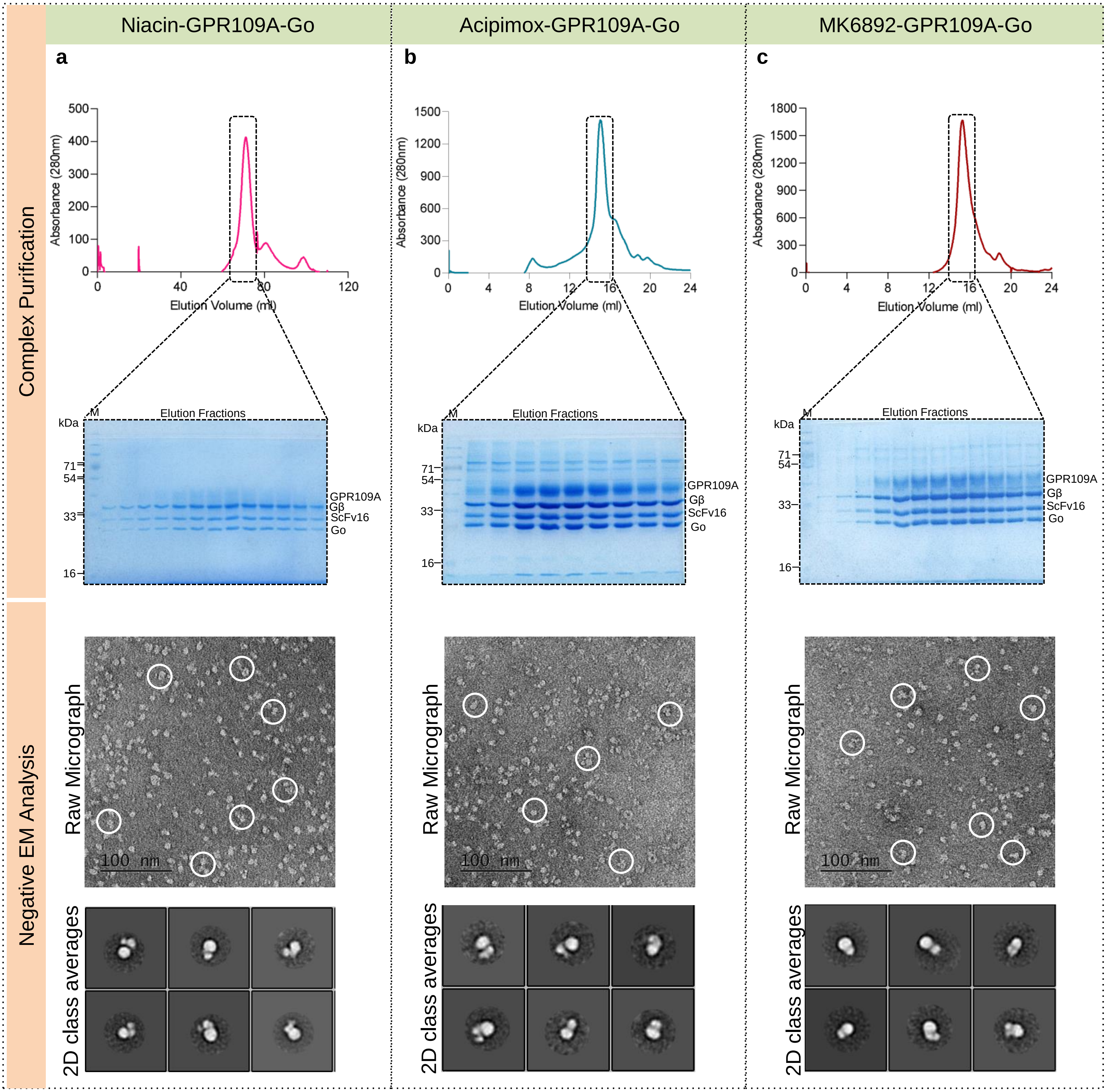

Supplementary Fig. 3. GPR109A-Go Complex Reconstitution and Visualization by Negative Staining EM. a-c, Size exclusion chromatogram, SDS-PAGE analysis and negative stain EM analysis of niacin-GRP109A-Go, acipimox-GPR109A-Go, and MK6892-GPR109A-Go complexes, respectively. Source data is provided as source data file.

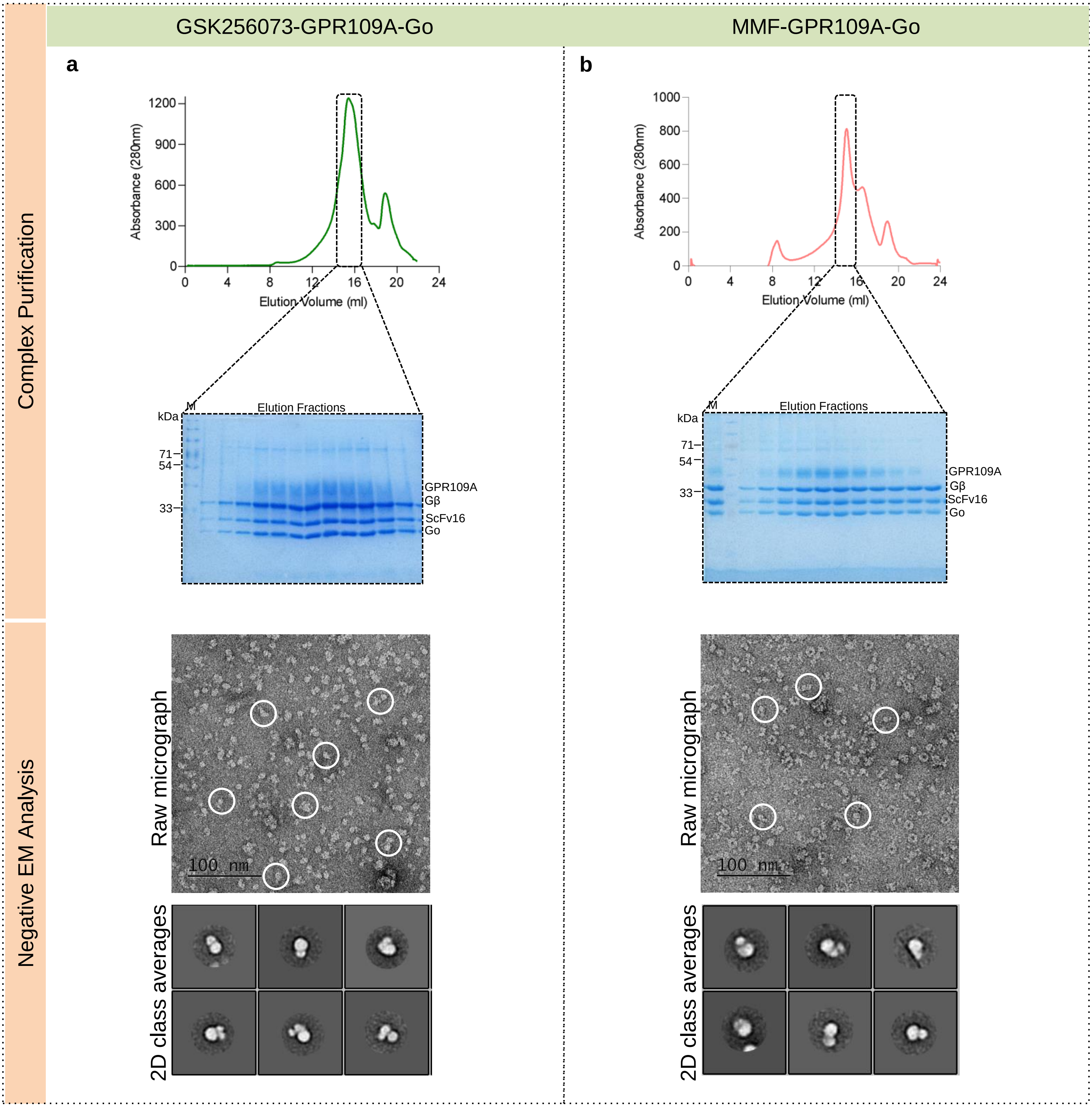

Supplementary Fig. 4. GPR109A-Go Complex Reconstitution and Visualization by Negative Staining EM. a, b, Size exclusion chromatogram, SDS-PAGE analysis and negative stain EM analysis of GSK256073-GRP109A-Go and MMF-GPR109A-Go complex, respectively. Source data is provided as source data file.

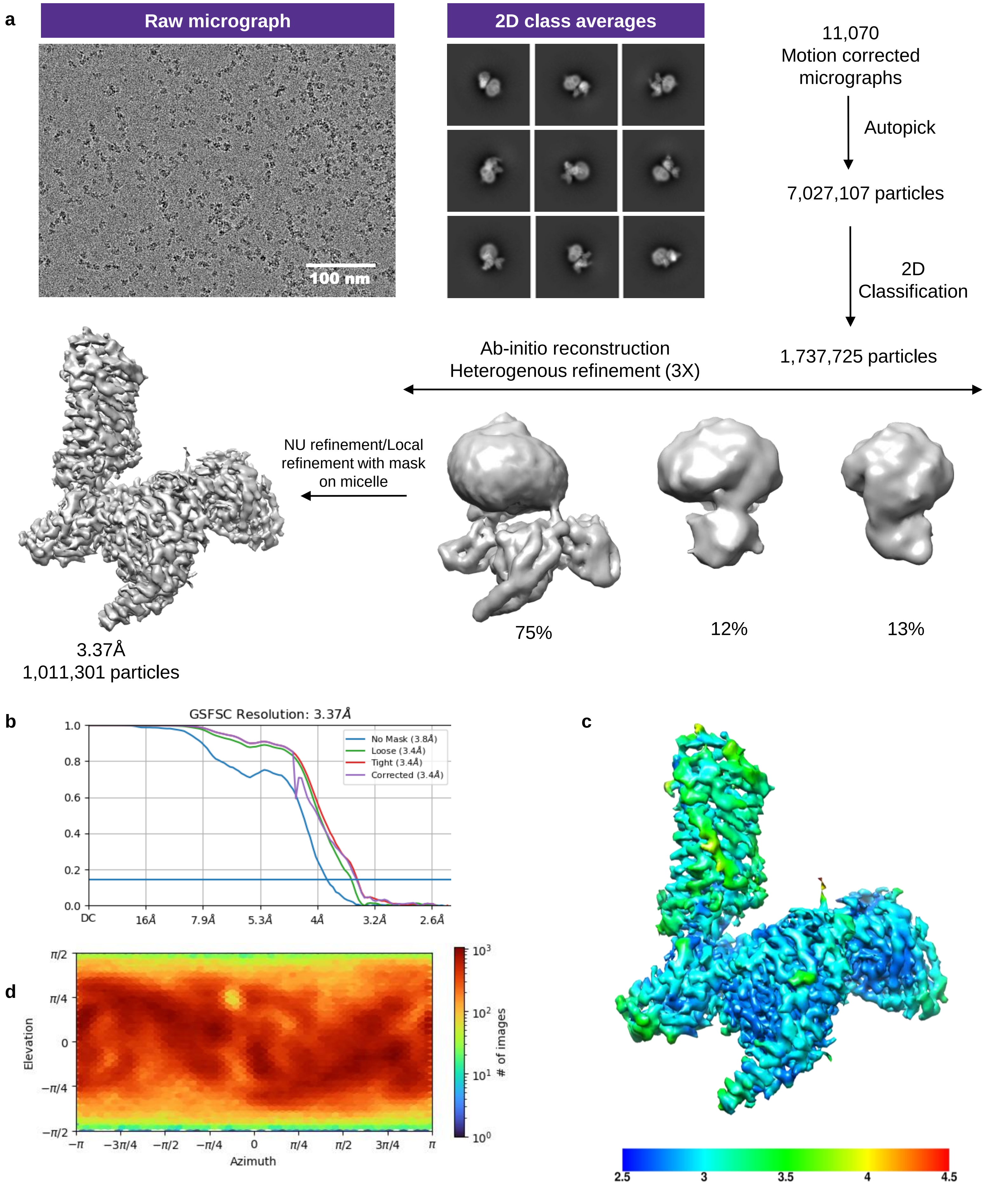

Supplementary Fig. 5. Cryo-EM data processing workflow of niacin-GPR109A-Go complex. a, Schematic representation of the cryo-EM data processing workflow. **b,** Gold standard fourier shell correlation curve (GFSC) at 0.143 threshold. **c,** Local resolution map of the 3D reconstruction (front view). **d,** Angular distribution of the particles used for final reconstruction.

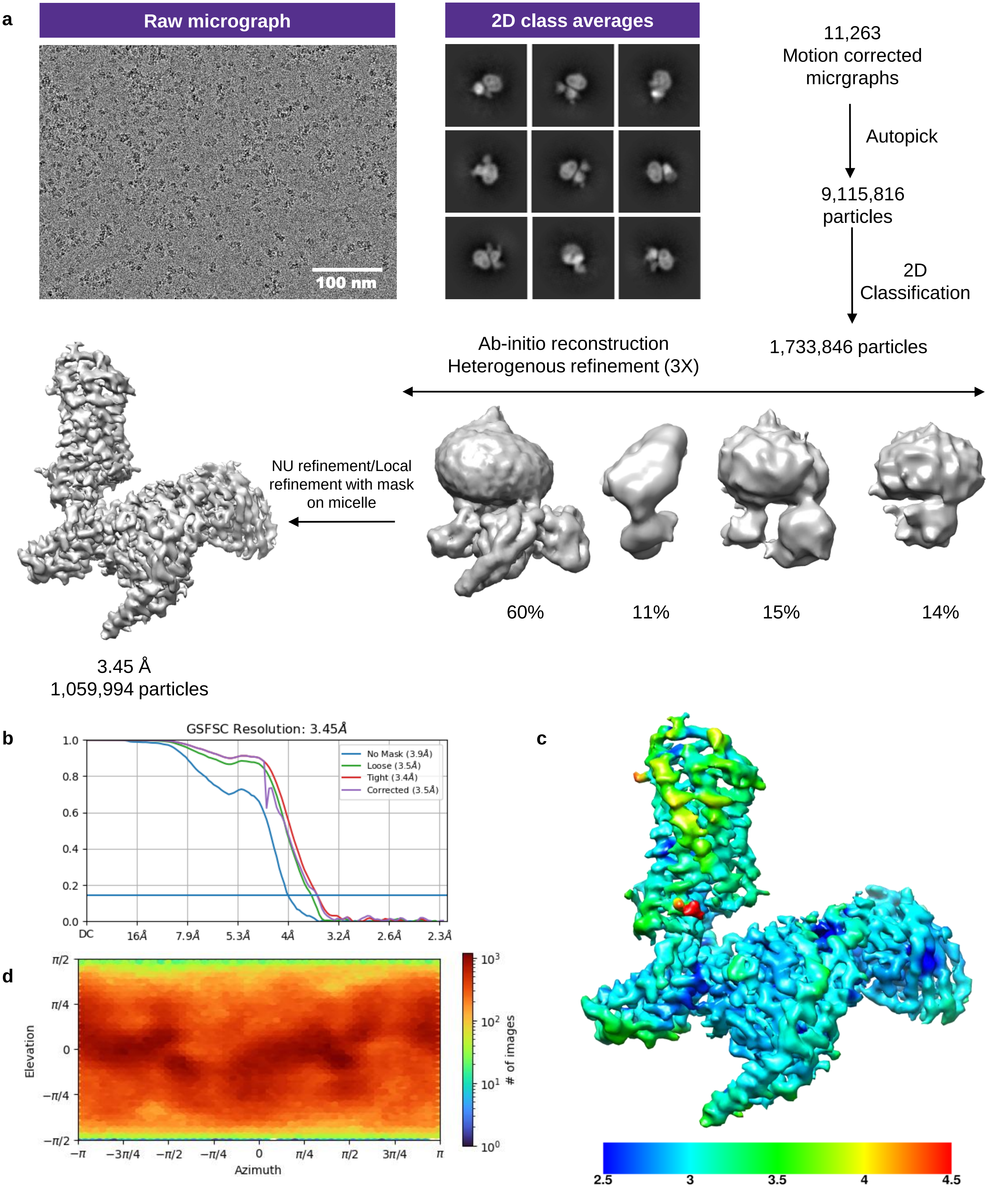

Supplementary Fig. 6. Data processing workflow of acipimox-GPR109A-Go complex. a, Flowchart of the cryo-EM data processing pipeline. **b,** Gold standard fourier shell correlation curve at 0.143 threshold. **c,** Local resolution map for the final 3D reconstruction (front view). **d,** Angular distribution of the particle set used for the final reconstruction.

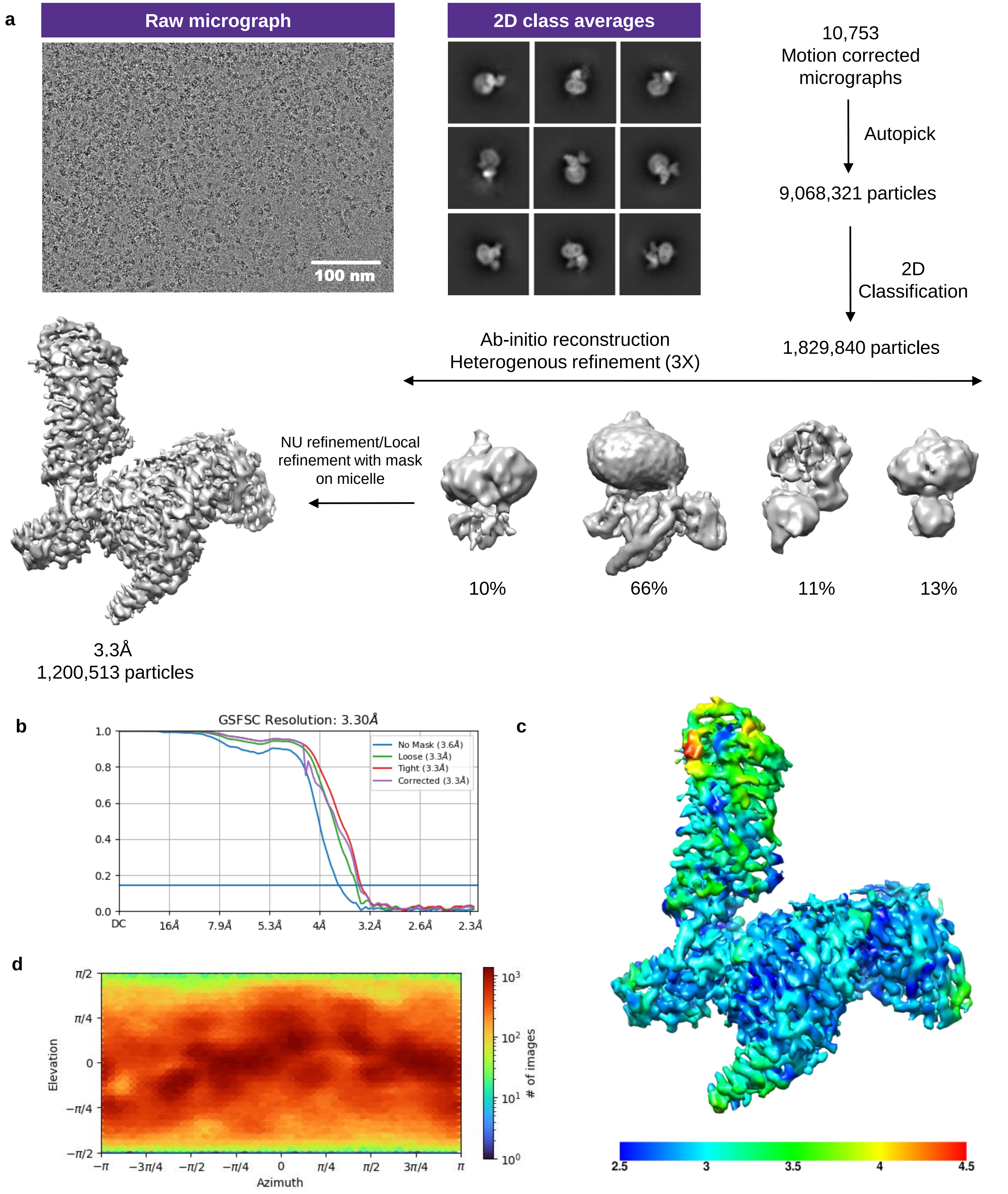

Supplementary Fig. 7. Cryo-EM reconstruction of MK6892-GPR109A-Go complex. a, Data processing workflow for reconstruction of MK6892-GPR109A-Go complex. **b,** Gold standard fourier shell correlation curve (GFSC) at a threshold of 0.143 indicates an overall resolution of 3.3Å. **c,** Local resolution map of the 3D reconstruction. **d,** Angular plot of the particles used for final 3D reconstruction of the complex.

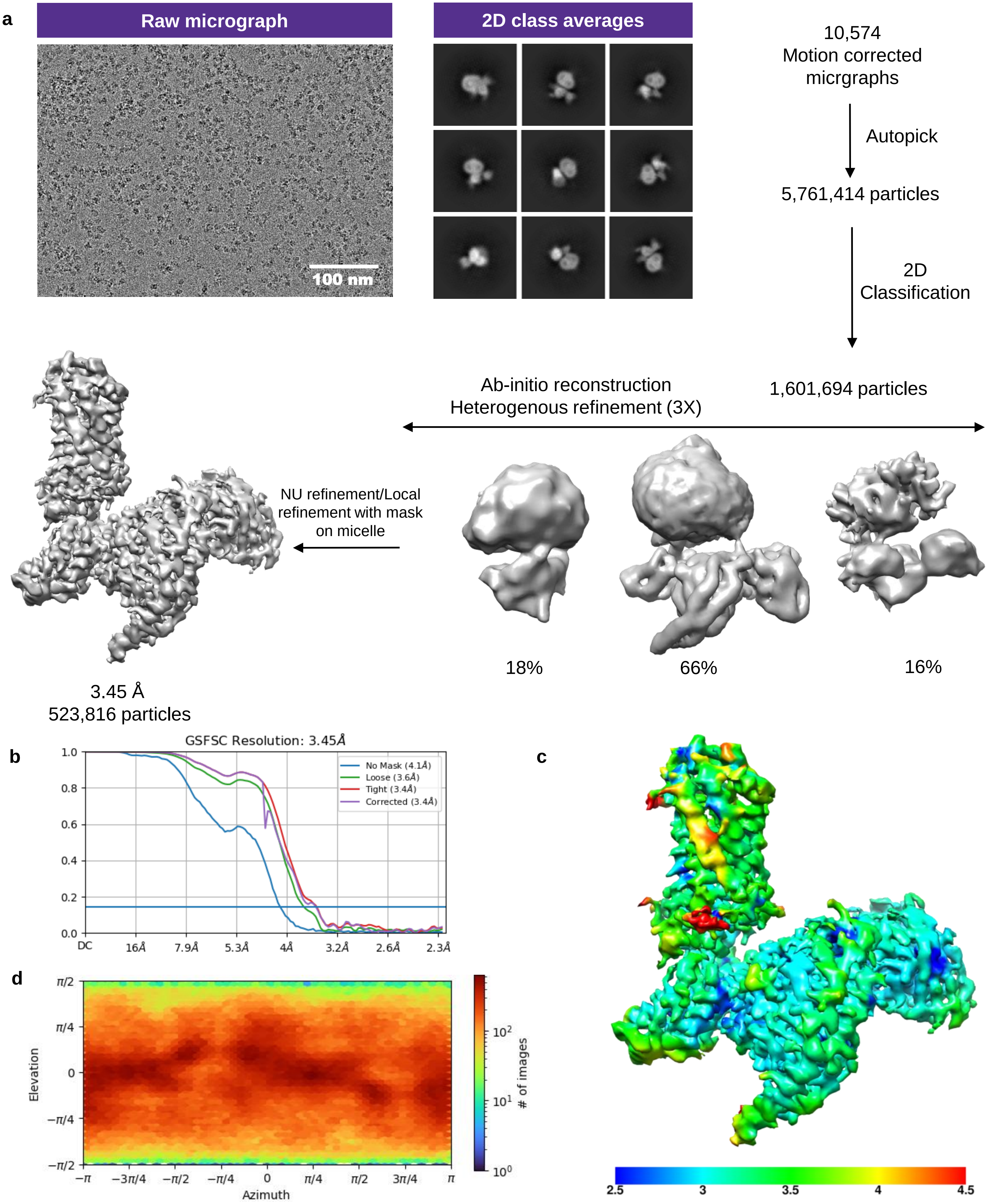

Supplementary Fig. 8. Data processing workflow of GSK256073-GPR109A-Go complex. a, Flowchart of the cryo-EM data processing pipeline. **b,** Gold standard fourier shell correlation curve at 0.143 threshold. **c,** Local resolution map for the final 3D reconstruction (front view). **d,** Angular distribution of the particle set used for the final reconstruction.

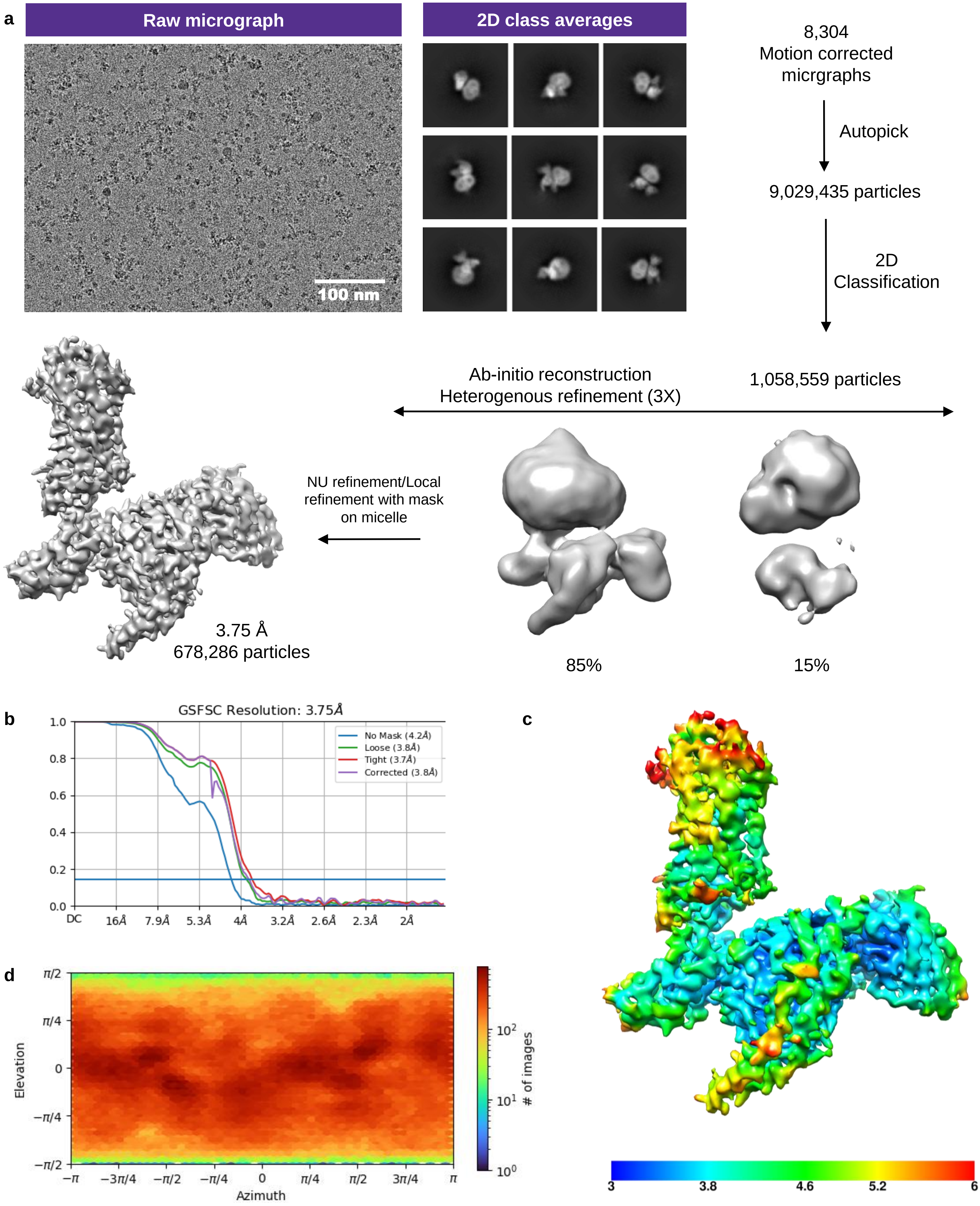

Supplementary Fig. 9. Data processing workflow of MMF-GPR109A-Go complex. a, Flowchart of the cryo-EM data processing pipeline. **b,** Gold standard fourier shell correlation curve at 0.143 threshold. **c,** Local resolution map for the final 3D reconstruction (front view). **d,** Angular distribution of the particle set used for the final reconstruction.

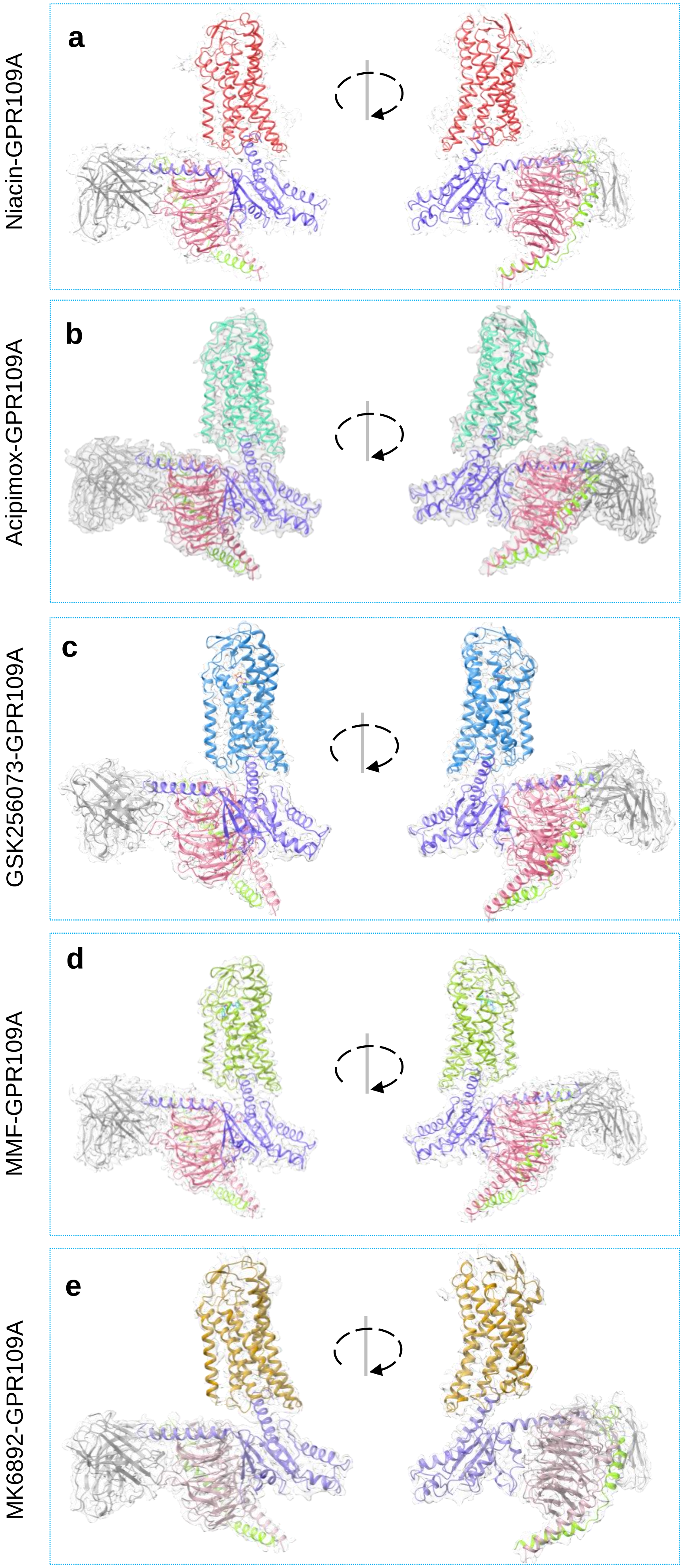

Supplementary Fig. 10. Ligand bound GPR109A model fit to map. a, b, c, d, e. Niacin, acipimox, GSK256073, MMF, and MK6892.

Data collection and processing						
		**Niacin-GPR109A**	**Acipimox-GPR109A**	**GSK256073-GPR109A**	**MK6892-GPR109A**	**MMF-GPR109A**
		PDB 8IY9	PDB 8JER	8IYW	PDB 8IYH	PDB 8JHN
		EMD-35817	EMD-36193	EMD-35831	EMD-35822	EMD-36280
Microscope		TFS Glacios	TFS Glacios	TFS Glacios	TFS Glacios	TFS Glacios
Camera		Gatan K3	Gatan K3	Gatan K3	Gatan K3	Gatan K3
Magnification		46,000x	46,000x	46,000x	46,000x	46,000x
Voltage (kV)		200	200	200	200	200
Defocus range (µm)		0.5-2.5	0.5-2.5	0.5-2.5	0.5-2.5	0.5-2.5
Exposure time (s)		4	4	4	4	4
Total dose (e^-/A^2)		55	52	55	55	55
Number of frames		40	40	40	40	40
Pixel size (Å)		0.878	0.878	0.878	0.878	0.878
Micrographs (no.)		11,070	11,263	10,574	10,753	8,304
Initial particles (no.)		70,27,107	91,15,816	57,61,414	90,68,321	90,29,435
Symmetry imposed		C1	C1	C1	C1	C1
Final particles (no.)		10,11,301	10,59,994	5,23,816	12,00,513	6,78,286
Map resolution (Å)		3.37	3.45	3.45	3.3	3.75
FSC threshold		0.143	0.143	0.143	0.143	0.143
Map resolution range (Å)		2.5-4.5	2.5-4.5	2.5-4.5	2.5-4.5	3.0–6.0
**Refinement**						
Initial model (PDB Code)		AlphaFold AF-Q8TDS4-F1	8IY9	8IY9	8IY9	8IY9
Model resolution (Å)		3.5	3.7	3.7	3.4	4
FSC threshold		0.5	0.5	0.5	0.5	0.5
Model resolution range (Å)		n/a	n/a	n/a	n/a	n/a
Map sharpening B-factor		-112.1	-133.2	-119.2	-158.5	-169.9
**Model composition**						
Non-hydrogen atoms		8,121	7,907	7,849	8,035	7,773
Protein residues		1,130	1,119	1,118	1,119	1,120
Ligand atoms		NIO=1	ACI=1	OKL=1	FI7=1	MMF=1
B factors (Å2)	Protein	34.58	49.71	89.51	50.04	43.82
	Ligand	64.14	89.29	77.83	66.6	89.4
**R.M.S. deviations**						
Bond length (Å)		0.006	0.005	0.006	0.006	0.005
Bond angle (º)		1.215	1.015	1.169	1.136	1.035
**Validation**						
Favored (%)		96.94	96.9	96.73	97.73	96.63
Allowed (%)		3.06	3.1	3.27	2.27	3.37
Disallowed (%)		0	0	0	0	0
MolProbity score		1.38	1.51	1.62	1.35	1.55
Clash Score		3.88	5.98	5.43	5.05	6.14
Poor rotamers (%)		1.07	0.14	1.45	1.08	0.6

Supplementary Fig. 11. Data collection and model refinement statistics: Data collection, processing and refinement statistics of niacin, acipimox, GSK256073, MK6892 and MMF bound GPR109A-Go complexes.

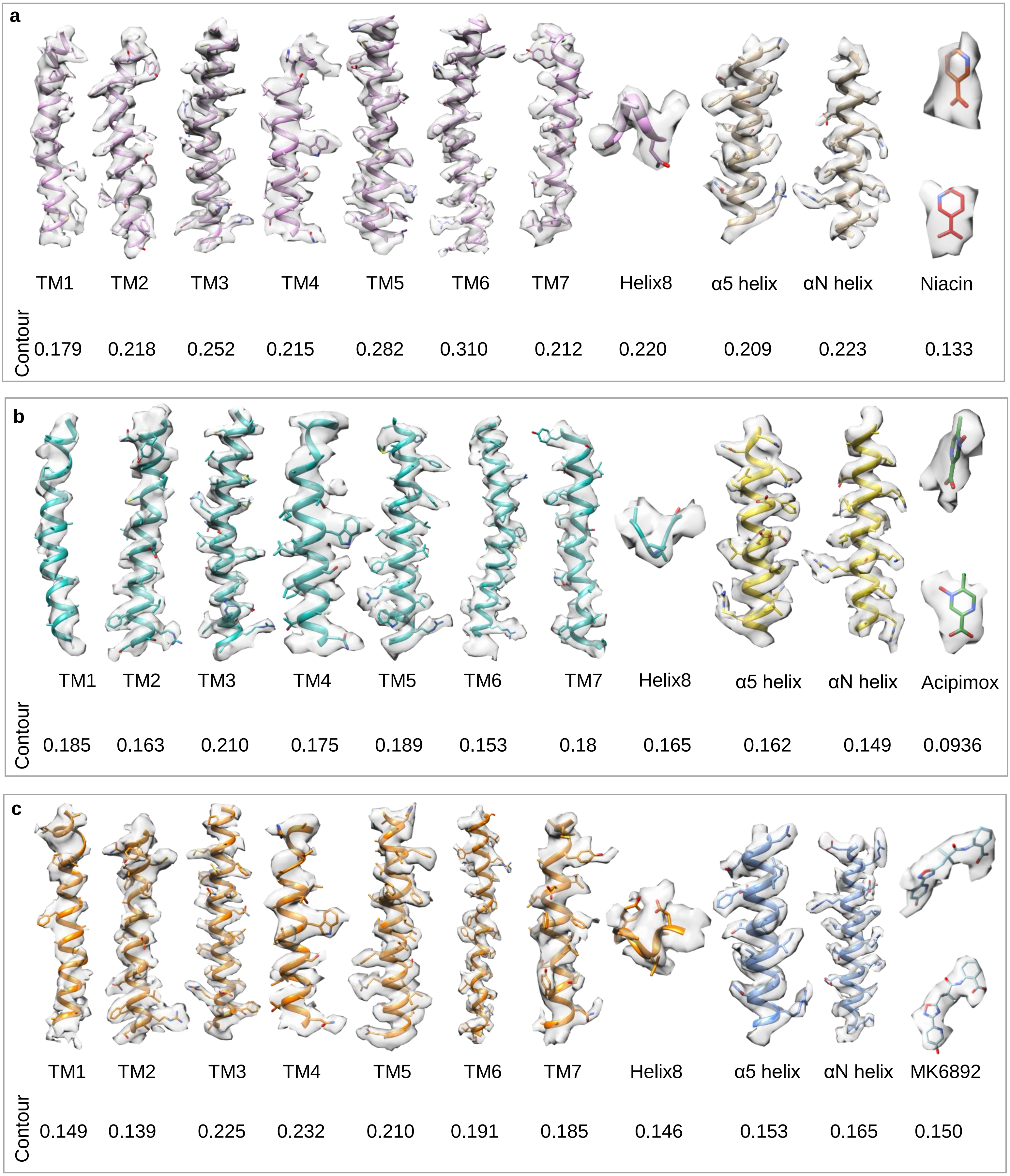

Supplementary Fig. 12. Representative EM density maps. a, EM densities for the niacin-GPR109A-Go structure (left to right): TM1 to TM7, helix 8, α5 helix, αN helix, and niacin. **b,** EM densities for the acipimox-GPR109A-Go structure (left to right): TM1 to TM7, α5 helix, αN helix, helix 8 and acipimox **c,** EM densities for the MK6892-GPR109A-Go structure (left to right): TM1 to TM7, helix8, α5 helix, αN helix, and MK6892.

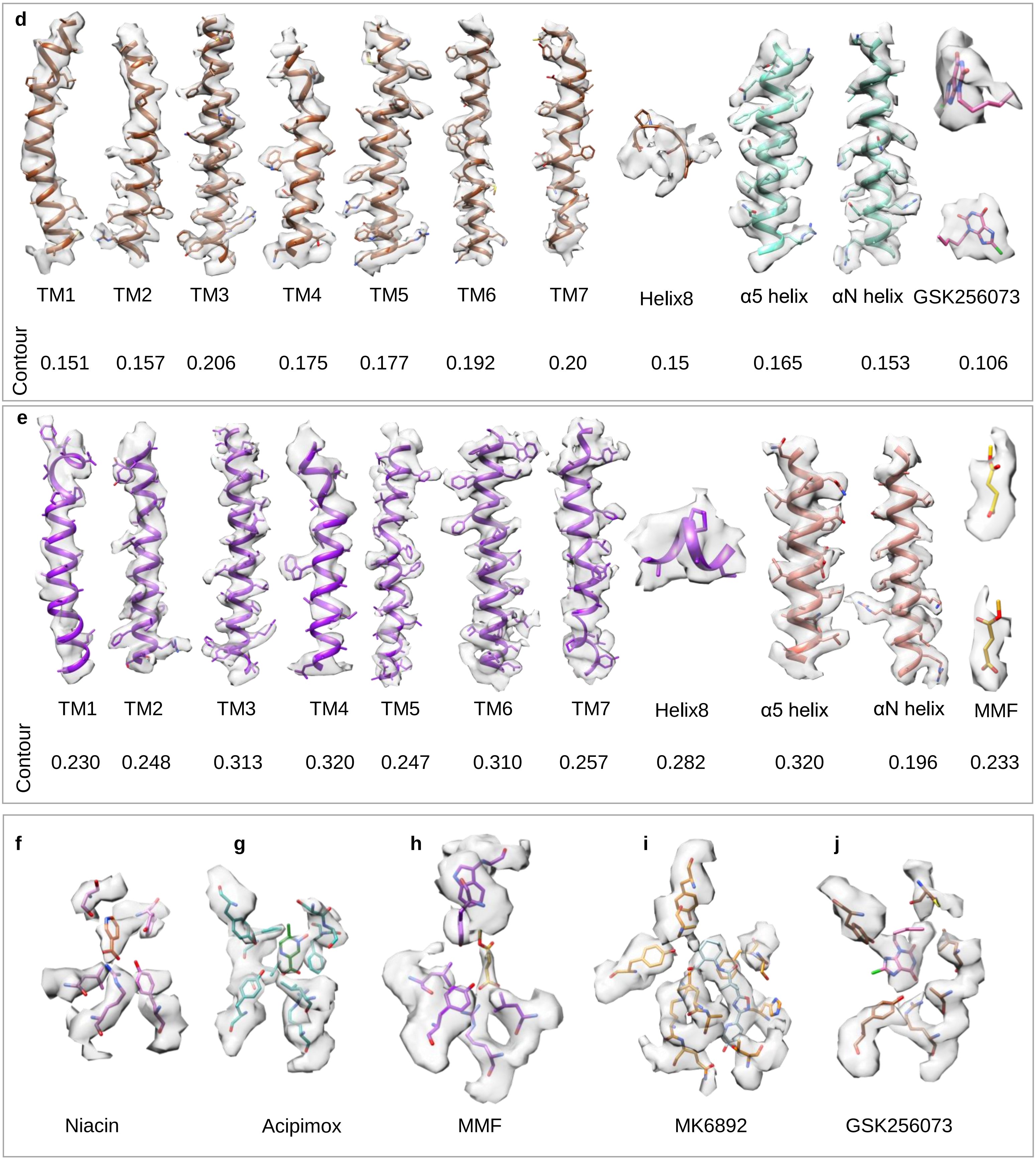

Supplementary Fig. 12 continue…, d, EM densities for the GSK256073-GPR109A-Go structure (left to right): TM1 to TM7, α5 helix, αN helix, helix 8 and GSK256073. **e,** EM densities for the MMF-GPR109A-Go structure (left to right): TM1 to TM7, helix8, α5 helix, αN helix, and MMF. **f, g, h, i, j.** The ligand binding site of niacin, acipimox, MMF, MK6892 and GSK256073 are displayed as a map fit with a stick model

Component	Total residues	Resolved residues	Total residues	Resolved residues
	Niacin-GPR109A-Go		Acipimox-GPR109A-Go	
**Ligand/Drug**	1	1	1	1
**GPR109A**	M1-P363	D8-F304	M1-P363	D8-F52<br>W59-H223<br>K225-N303
**miniGαo**	M1-H57<br>T172-Y366	S6-I55<br>T183-D230<br>H245-Y354	M1-H57<br>T172-Y366	S6-M53<br>T183-D230<br>H245-Y354
**Gβ**	M1-N340	E3-N340	M1-N340	E3-N340
**Gγ**	M1-L71	A7-R62	M1-L71	S8-R62
**ScFv16**	D1-K248	D1-R72<br>K76-S120<br>G135-K248	D1-K248	D1-R72<br>K76-S120<br>G135-K248

Component	Total residues	Resolved residues	Total residues	Resolved residues
	MK6892-GPR109A-Go		GSK256073-GPR109A-Go	
**Ligand/Drug**	1	1	1	1
**GPR109A**	M1-P363	H9-F54<br>W59-S300	M1-P363	H9-H55<br>S58-F301
**miniGαo**	M1-H57<br>T172-Y366	S6-M53<br>T183-D230<br>H245-Y354	M1-H57<br>T172-Y366	S6-I149<br>T183-D230<br>H245-Y354
**Gβ**	M1-N340	E3-N340	M1-N340	E3-N340
**Gγ**	M1-L71	A7-R62	M1-L71	S8-R62
**ScFv16**	D1-K248	D1-R72<br>K76-S120<br>G135-K248	D1-K248	D1-S120<br>S136-L247

Component	Total residues	Resolved residues
	MMF-GPR109A-Go	
**Ligand/Drug**	1	1
**GPR109A**	M1-P363	D8-F52, K60-N137, S140-F305
**miniGαo**	M1-H57<br>T172-Y366	S6-M53, T183-D230, H245-Y354
**Gβ**	M1-N340	E3-N340
**Gγ**	M1-L71	A7-R62
**ScFv16**	D1-K248	D1-R72, K76-S120, G135-K248

Supplementary Fig. 13. Residues resolved in the structures of niacin, acipimox, MK6892, GSK256073 and MMF bound GPR109A-Go complexes.

a

Chain R (GPR109A)	Distance (Å)	Chain N (Niacin)
Leu83 (TM2)	3.83	Nio
Tyr87 (TM2)	3.60	Nio
Leu104 (TM3)	3.64	Nio
Leu107 (TM3)	3.09	Nio
Arg111 (TM3)	2.69	Nio
Ser178 (ECL2)	3.67	Nio
Ser179 (ECL2)	3.36	Nio
Phe180 (ECL2)	3.61	Nio
Phe277 (TM7)	3.90	Nio
Leu280 (TM7)	3.72	Nio
Tyr284 (TM7)	2.74	Nio

b

Chain R (GPR109A)	Distance (Å)	Chain R Acipimox
Tyr87 (TM2)	3.26	OJX
Leu104 (TM3)	3.60	OJX
Leu107 (TM3)	3.11	OJX
Arg111(TM3)	2.58	OJX
Cys177 (ECL2)	3.70	OJX
Ser178 (ECL2)	3.19	OJX
Ser179 (ECL2)	2.56	OJX
Phe180 (ECL2)	3.20	OJX
Phe277 (TM7)	3.34	OJX
Leu280 (TM7)	3.12	OJX
Tyr284 (TM7)	2.64	OJX

c

Chain D (GPR109A)	Distance (Å)	Chain Z (MK6892)
Leu83 (TM2)	3.53	FI7
Tyr87 (TM2)	3.42	FI7
Leu107 (TM3)	3.05	FI7
Ala108 (TM3)	3.45	FI7
Arg111 (TM3)	2.76	FI7
Gln112 (TM3)	3.03	FI7
Thr159 (TM4)	3.31	FI7
Ser179 (ECL2)	3.22	FI7
Phe180 (ECL2)	3.42	FI7
His189 (TM5)	2.96	FI7
Met192 (TM5)	3.69	FI7
Tyr284(TM7)	3.08	FI7

d

Chain R (GPR109A)	Distance (Å)	Chain Z (GSK256073)
Tyr87 (TM2)	2.67	OKL
Leu104 (TM3)	3.39	OKL
Leu107 (TM3)	3.24	OKL
Arg111 (TM3)	3.31	OKL
Cys177 (ECL2)	3.24	OKL
Ser178 (ECL2)	3.49	OKL
Ser179 (ECL2)	3.29	OKL
Phe277 (TM7)	3.86	OKL
Leu280 (TM7)	3.85	OKL
Tyr284 (TM7)	3.56	OKL

e

Chain D (GPR109A)	Distance (Å)	Chain C (MMF)
Tyr87 (TM2)	2.87	UR9
Trp91 (ECL1)	3.13	UR9
Leu107 (TM3)	3.29	UR9
Arg111 (TM3)	2.86	UR9
Ser178 (ECL2)	3.81	UR9
Leu280 (TM7)	3.47	UR9
Tyr284 (TM7)	2.77	UR9

Supplementary Fig. 14. Interaction of niacin, acipimox, MK6892, GSK256073 and MMF with GPR109A. a-e, List of interaction between niacin, acipimox, MK6892, GSK256073 and MMF with GPR109A (Residues within 4 Ǻ are shown). Interacting residues and interaction distances listed in the table are generated using the software PDBsum.

a

Chain R (GPR109A)	Distance (Å)	Chain A (Gαo)
Ser62 (TM2)	Gly350 (2.93)	Gly350
Arg125 (TM3)	Cys351 (3.40)	Cys351
Arg128 (TM3)	Asn347 (3.01)	Asn347
Val129 (TM3)	Leu348 (3.45)	Leu348
Pro132 (ICL2)	Ile344 (3.56)	Ile344
His133 (ICL2)	Leu195 (3.68), Thr340 (3.55)	Leu195, Thr340
Leu215 (TM5)	Ile344 (3.72)	Ile344
Arg218 (TM5)	Thr340 (2.84), Asp341 (2.65), Ile344 (3.59)	Thr340, Asp341, Ile344
Met220 (ICL3)	Ile344 (3.52)	Ile344
His223 (ICL3)	Glu318 (3.29)	Glu318
Lys225 (TM6)	Tyr354 (3.23)	Tyr354
Ile226 (TM6)	Leu348 (3.75), Tyr354 (3.63)	Leu348, Tyr354
Arg228 (TM6)	Tyr354 (2.94)	Tyr354
Ala229 (TM6)	Leu353 (3.36)	Leu353
Phe232 (TM6)	Leu353 (3.79)	Leu353
Ile233 (TM6)	Leu353 (3.76)	Leu353
Ser297 (H8)	Gly352 (3.07)	Gly352
Ser298 (H8)	Gly352 (3.07)	Gly352
Pro299 (H8)	Tyr354 (3.76)	Tyr354

b

Chain R (GPR109A)	Distance (Å)	Chain A (Gαo)
Ser62 (TM2)	Gly350 (2.89), Cys351 (3.51)	Gly350, Cys351
Asp124 (TM3)	Cys351 (3.85)	Cys351
Arg125 (TM3)	Leu353 (3.49)	Leu353
Arg128 (TM3)	Asn347 (3.44), Cys351 (2.83)	Asn347, Cys351
Val129 (TM3)	Leu348 (3.59)	Leu348
Pro132 (ICL2)	Thr340 (3.60), Ile344 (3.64)	Ithr340, Ile344
His133 (ICL2)	Leu195 (3.49), Phe336 (3.84), Thr340 (3.37), Ile343 (3.83)	Leu195, Phe336, Thr340, Ile343
Leu215 (TM5)	Ile344 (3.75)	Ile344
Arg218 (ICL3)	Thr340 (3.38), Asp341 (3.05)	Thr340, Asp341
Met220 (ICL3)	Ile344 (3.48)	Ile344
His223 (TM6)	Glu318 (2.82)	Glu318
Lys225 (TM6)	Tyr354 (3.73)	Tyr354
Ile226 (TM6)	Tyr354 (3.59)	Tyr354
Arg228 (TM6)	Tyr354 (3.36)	Tyr354
Ala229 (TM6)	Leu353 (3.45)	Leu353
Ser297	Gly352 (3.23)	Gly352
Ser298	Gly352 (3.20)	Gly352

Supplementary Fig. 15. Interaction of Go with GPR109A. a, b, List of GPR109A-Go interactions in the structures of niacin, acipimox, MK6892, GSK256073 and MMF-GPR109A-Go (Residues within 4 Å radius are shown). Receptor and G-protein interacting residues in the solved structures and their corresponding interacting distances mentioned in the table are generated using the software PDBsum.

c

Chain D (GPR109A)	Distance (Å)	Chain B (GαO)
Lys60 (TM2)	Gly350 (3.28)	Gly350
Ser62 (TM2)	Gly350 (2.88), Cys351 (3.39)	Gly350, Cys351
Arg125 (TM3)	Leu353 (3.41)	Leu353
Arg128 (TM3)	Asn347 (3.36), Cys351 (3.67)	Asn347, Cys351
Val129 (TM3)	Leu348 (3.63)	Leu348
Pro132 (ICL2)	Ile343 (3.88), Ile344 (3.49)	Ile343, Ile344
His133 (ICL2)	Leu195 (3.50), Thr340 (2.75), Ile343 (3.54)	Leu195, Thr340, Ile343
Arg218 (TM5)	Asp337 (3.88), Thr340 (3.38), Asp341 (2.96)	Asp337, Thr340, Asp341
Met220 (ICL3)	Ile344 (3.42)	Ile344
His223 (ICL3)	Glu318 (2.82)	Glu318
Lys225 (TM6)	Tyr354 (3.48)	Tyr354
Ile226 (TM6)	Tyr354 (3.54)	Tyr354
Ala229 (TM6)	Leu353 (3.47)	Leu353
Ile233 (TM6)	Leu353 (3.79)	Leu353
Ser297 (H8)	Leu353 (3.01), Tyr354 (3.65)	Leu353, Tyr354
Ser298 (H8)	Gly352 (3.37)	Gly352
Pro299 (H8)	Tyr354 (3.43)	Tyr354

d

Chain R (GPR109A)	Distance (Å)	Chain B (Gαo)
Arg125 (TM3)	Leu353 (3.40)	Leu353
Arg128 (TM3)	Asn347 (3.01), Gly350 (3.65), Cys351 (3.47)	Asn347, Gly350, Cys351
Val129 (TM3)	Ile344 (3.84), Leu348 (3.57)	Ile344, Leu348
Pro132 (ICL2)	Ile344 (3.64), Asn347 (3.39)	Ile344, Asn347
His133 (ICL2)	Leu195 (3.86), Thr340 (3.39)	Leu195, Thr340
Lys138 (ICL2)	Ala31 (3.80)	Ala31
Arg218 (TM5)	Thr340 (3.31), Asp341 (3.10), Ile344 (3.71)	Thr340, Asp341, Ile344
Met220 (ICL3)	Ile344 (3.52)	Ile344
His223 (ICL3)	Glu318 (2.55)	Glu318
Lys225 (TM6)	Tyr354 (3.49)	Tyr354
Ile226 (TM6)	Leu348 (3.80), Tyr354 (3.42)	Leu348, Tyr354
Ala229 (TM6)	Leu353 (3.39)	Leu353
Ile233 (TM6)	Leu353 (3.72)	Leu353
Ser298 (H8)	Gly352 (2.76), Leu353 (3.31)	Gly352, Leu353

Supplementary Fig. 15 continue…c, d.

e

Chain D (GPR109A)	Distance (Å)	Chain A (Gαo)
Ser62 (TM2)	Gly350 (3.59), Cys351 (3.60)	Gly350, Cys351
Arg125 (TM3)	Cys351 (3.84), Leu353 (3.37)	Cys351, Leu353
Arg128 (TM3)	Asn347 (3.45), Cys351 (3.22)	Asn347, Cys351
Val129 (TM3)	Ile344 (3.78), Leu348 (3.69)	Ile344, Leu348
Pro132 (ICL2)	Ile343 (3.76), Ile344 (3.55)	Ile343, Ile344
His133 (ICL2)	Thr340 (3.25), Ile343 (3.59)	Thr340, Ile343
Arg218 (ICL3)	Asp337 (3.59), Thr340 (2.59), Asp341 (3.50)	Asp337, Thr340, Asp341
Met220 (ICL3)	Asp341 (3.11), Ile344 (3.43)	Asp341, Ile344
Lys225 (TM6)	Tyr354 (3.54)	Tyr354
Ile226 (TM6)	Tyr354 (3.55)	Tyr354
Ala229 (TM6)	Leu353 (3.49)	Leu353
Ser297	Gly352 (3.24), Leu353 (3.59)	Gly352, Leu353
Ser298	Gly352 (3.33)	Gly352

Supplementary Fig. 15 continue…e, f. List of GPR109A-Go interactions in the structures of niacin, acipimox, MK6892, GSK256073 and MMF-GPR109A-Go (Residues within 4 Å radius are shown). Receptor and G-protein interacting residues in the solved structures and their corresponding interacting distances mentioned in the table are generated using the software PDBsum.

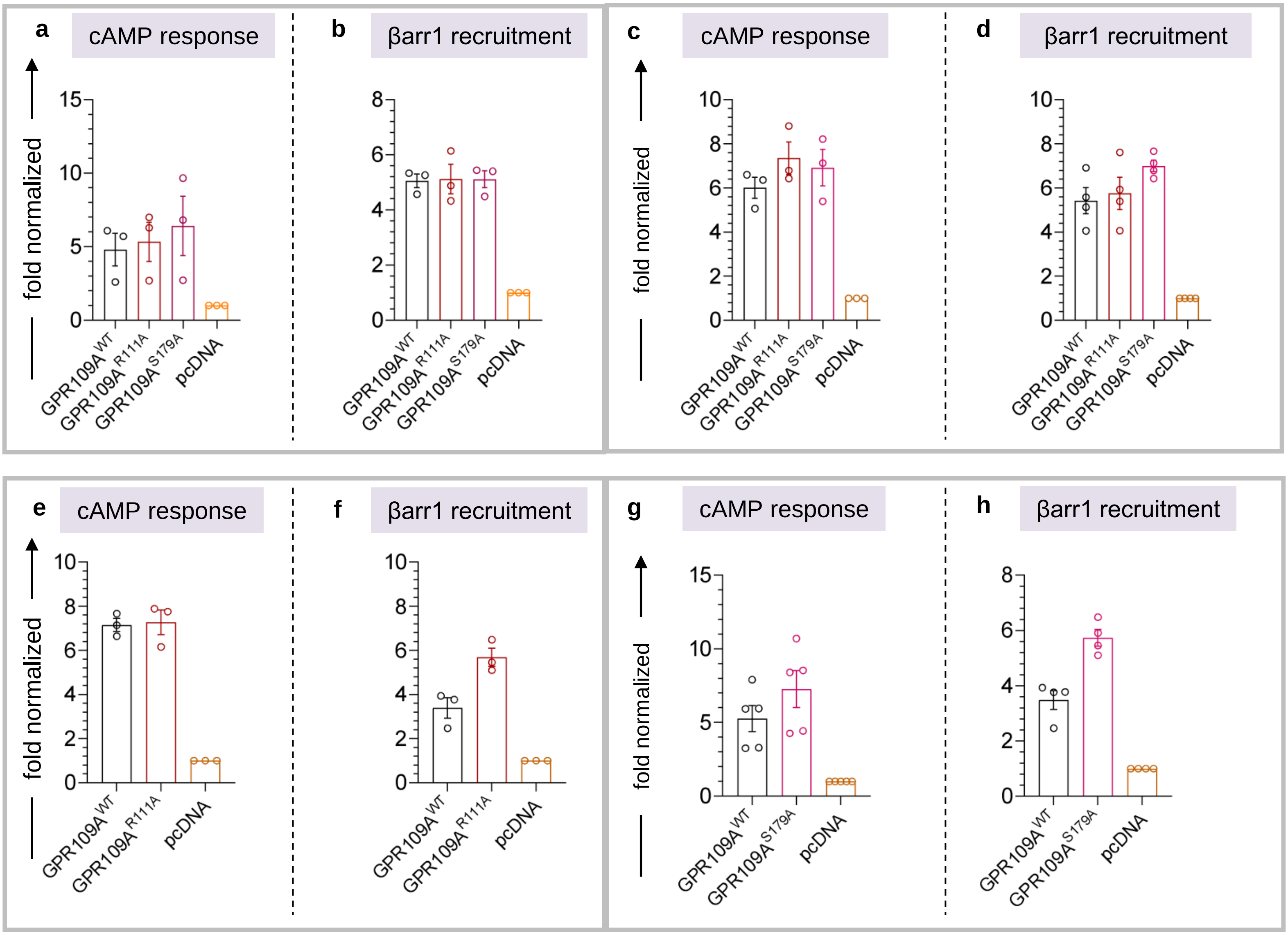

Supplementary Fig. 16. Surface expression of GPR109A^{WT} and mutants in various assays. a, b, Surface expression of GPR109A^{WT}, GPR109A^{R111A}, and GPR109A^{S179A} in single point cAMP response assay and βarr1 recruitment assay in response to niacin, acipimox, GSK256073, MMF, and MK6892 was measured using whole cell based surface ELISA (mean±SEM; n=3 independent experiments; normalized as fold over pcDNA. **c, d,** Surface expression of the indicated constructs in cAMP response assay (panel c) and βarr1 recruitment (panel d) dose response assay in response to niacin (mean±SEM; n=3-4 independent experiments; i.e., for cAMP response: n=3 and for βarr recruitment: n=4; normalized as fold over pcDNA). **e, f,** Surface expression of GPR109^{WT} and GPR109A^{R111A} in cAMP response assay (panel e) and βarr1 recruitment assay (panel f) in response to MK6892 (mean±SEM; n=3 independent experiments; normalized as fold over pcDNA). **g, h,** Surface expression of the GPR109A^{WT} and GPR109A^{S179A} in cAMP response assay (panel g) and βarr1 recruitment assay (panel h) in response to GSK256073 (mean±SEM; n=4-5 independent experiments; i.e., for cAMP response: n=5 and for βarr recruitment: n=4; normalized as fold over pcDNA). Source data is provided as source data file.

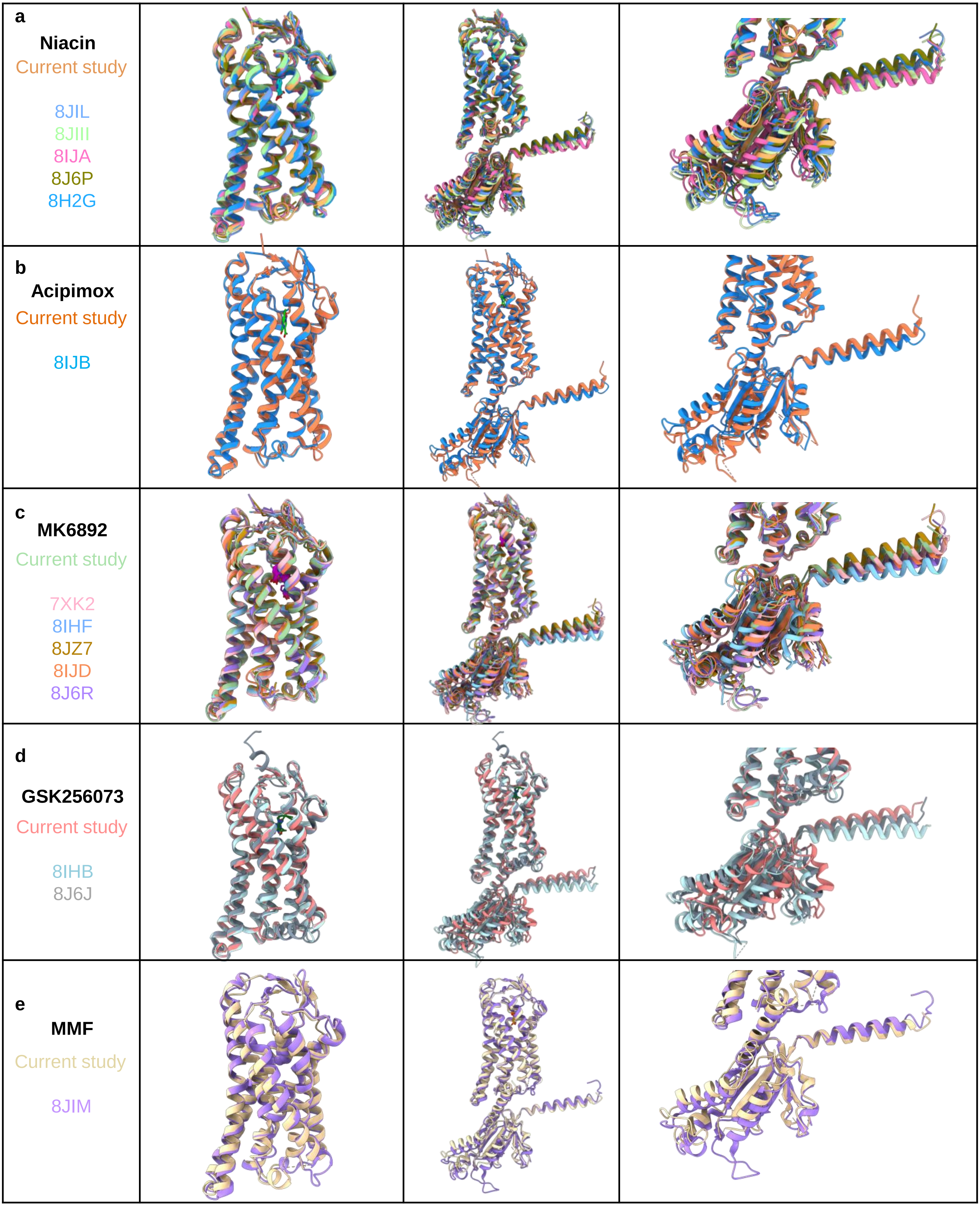

Supplementary Fig. 17. Comparative analysis of ligand bound GPR109A structures. a-e, Superimposition of niacin (**a**), acipimox (**b**) MK6892 (**c**), GSK256073 (**d**), and MMF (**e**), GPR109A-Go structures with already published structures of niacin (PDB ID: 8JIL, 8JII, 8IJA, 8J6P, 8H2G); acipimox (PDB ID: 8IJB); MK6892 (PDB ID: 7XK2, 8IHF, 8JZ7, 8IJD, 8J6R); GSK256073 (PDB ID: 8IHB, 8J6J); MMF (PDB ID: 8JIM), respectively.

Supplementary Table 1: List of the primers used in the study.		
**Construct**	**Primer**	**Sequence**
GPR109A_pcDNA3.1	GPR109A_pcDNA3.1_Fw	CTAGCTAGCATGGGCAAGACCATCATC
	GPR109A_pcDNA3.1_Rv	CGGGGTACCTTATTATTATGGGGAAGTAGGTCC
GPR109A_cSmBiT	GPR109A_cSmBiT_Fw	CGGGGTACCGAGGAGATCTGCCACCATGG
	GPR109A_cSmBiT_Rv	TCCCCCGGGTGGGGAAGTAGGTCCGAG
GPR109A(R111A)	GPR109A(R111A)_Fw	GGCCATGAACGCCCAGGGATCAATC
	GPR109A(R111A)_Rv	AGCATGAACAGCATCAGG
GPR109A(S179A)	GPR109A(S179A)_Fw	CCTGTGCTCTGCCTTCTCCATCTGC
	GPR109A(S179A)_Rv	TTAGCGCCACCGTTCTGG

Source Data File: Supplementary Figure 3a.

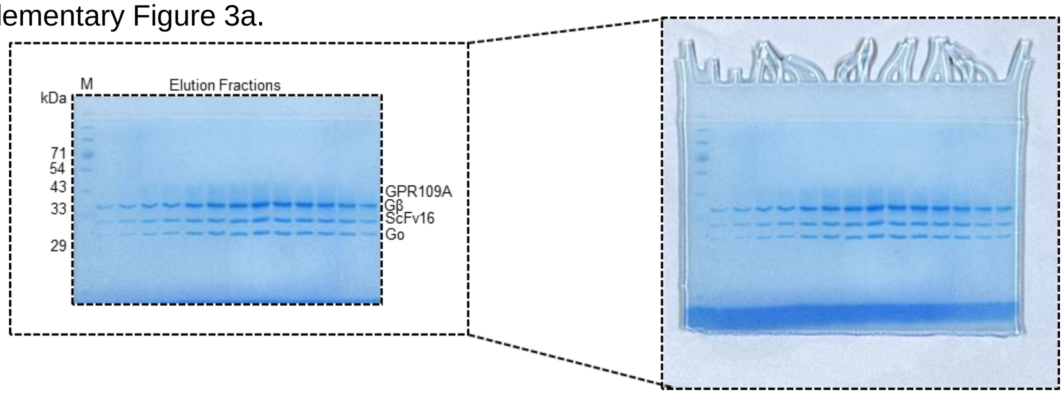

Source Data File: Supplementary Figure 3b.

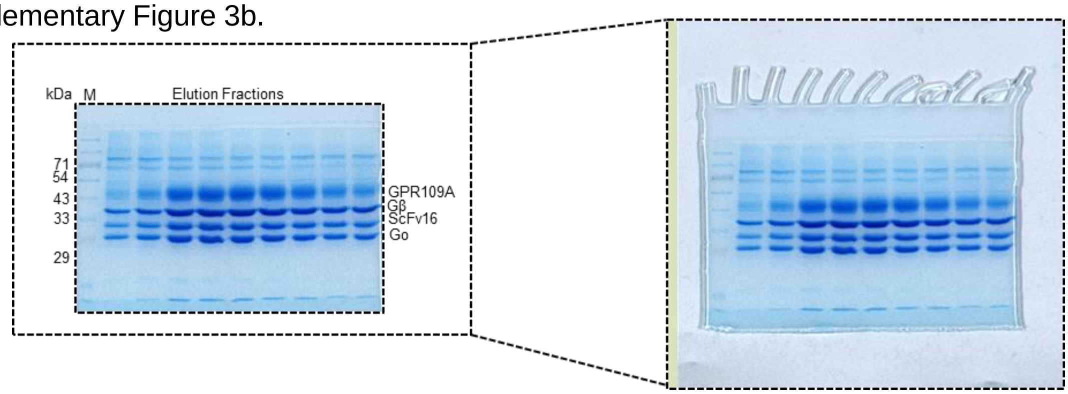

Source Data File: Supplementary Figure 3c.

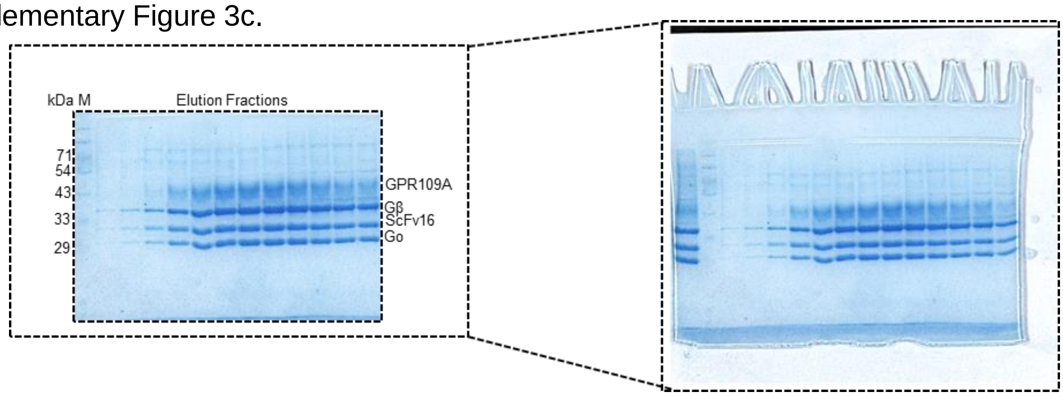

Source Data File: Supplementary Figure 4a.

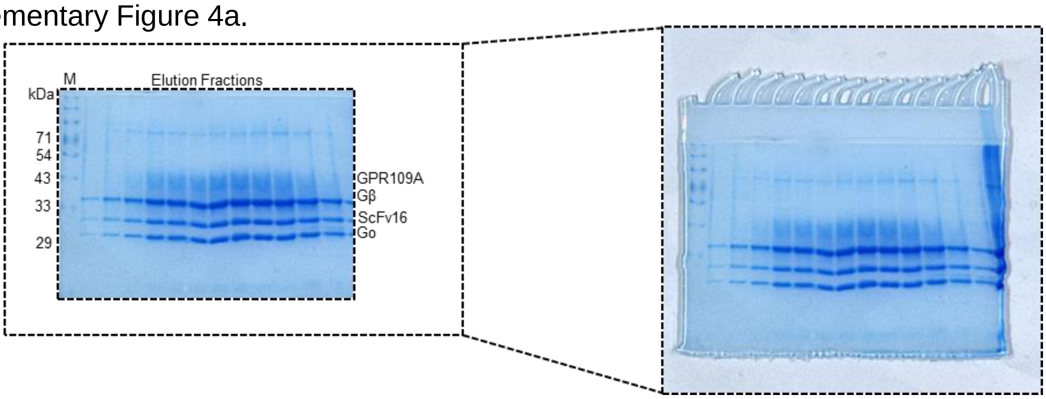

Source Data File: Supplementary Figure 4b.

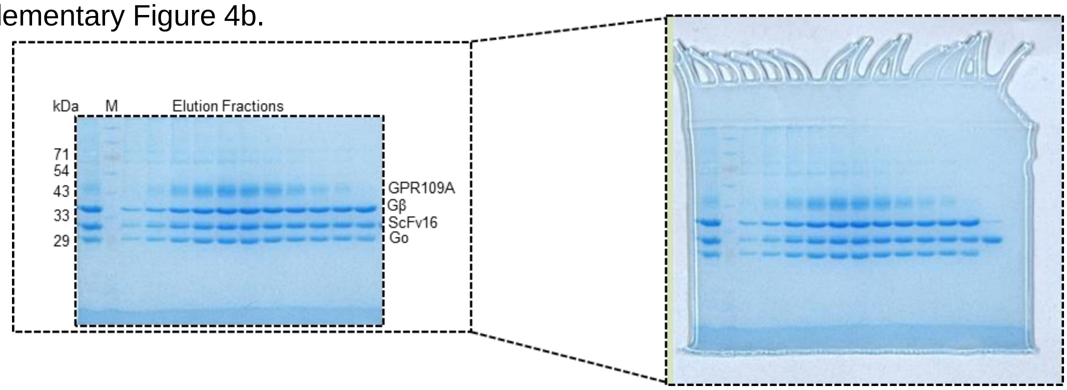

Supplement: Supplementary file 1 — Supplementary Information [file 41467_2024_46239_MOESM1_ESM.pdf]
