# Supplementary material for: Structure-guided engineering of biased-agonism in the human niacin receptor via single amino acid substitution
Source: Nat Commun. 2024 Mar 2;15:1939. doi: 10.1038/s41467-024-46239-2 (PMC10908815; doi:10.1038/s41467-024-46239-2)
Supplement: Supplementary file 3 — Reporting Summary [file 41467_2024_46239_MOESM3_ESM.pdf]

nature portfolio

Corresponding author(s): Arun K Shukla

Last updated by author(s): Feb 5, 2024

Reporting Summary

Nature Portfolio wishes to improve the reproducibility of the work that we publish. This form provides structure for consistency and transparency in reporting. For further information on Nature Portfolio policies, see our Editorial Policies and the Editorial Policy Checklist.

Statistics

For all statistical analyses, confirm that the following items are present in the figure legend, table legend, main text, or Methods section.

n/a	Confirmed	
☐	☒	The exact sample size (*n*) for each experimental group/condition, given as a discrete number and unit of measurement
☐	☒	A statement on whether measurements were taken from distinct samples or whether the same sample was measured repeatedly
☒	☐	The statistical test(s) used AND whether they are one- or two-sided<br>*Only common tests should be described solely by name; describe more complex techniques in the Methods section.*
☒	☐	A description of all covariates tested
☐	☒	A description of any assumptions or corrections, such as tests of normality and adjustment for multiple comparisons
☐	☒	A full description of the statistical parameters including central tendency (e.g. means) or other basic estimates (e.g. regression coefficient) AND variation (e.g. standard deviation) or associated estimates of uncertainty (e.g. confidence intervals)
☒	☐	For null hypothesis testing, the test statistic (e.g. *F*, *t*, *r*) with confidence intervals, effect sizes, degrees of freedom and *P* value noted<br>*Give P values as exact values whenever suitable.*
☒	☐	For Bayesian analysis, information on the choice of priors and Markov chain Monte Carlo settings
☒	☐	For hierarchical and complex designs, identification of the appropriate level for tests and full reporting of outcomes
☒	☐	Estimates of effect sizes (e.g. Cohen's *d*, Pearson's *r*), indicating how they were calculated

Our web collection on statistics for biologists contains articles on many of the points above.

Software and code

Policy information about availability of computer code

Data collection	Omega series microplate plate reader, BMG LABTECH, VictorX4 Perkinelmer plate reader, Bio-rad ChemiDoc Imaging System, imageQunatLAS 500. Cryo-EM data was collected on TFS Glacios (200kV) using SerialEM, UCSF chimeraX1.5, AlphaFold2
Data analysis	Following tools and softwares were used for data analysis:<br><br>Negative staining: RELION 3.1.2<br><br>CryoEM: cryoSPARC v4.0, Relion 3.1.2, Phenix 1.20, COOT 0.9.6, UCSF Chimera-1.15, UCSF ChimeraX-1.5, MolProbity (v4.5).<br><br>Biochemical and cellular assays: Omega series microplate plate reader.<br><br>For Cellular data analysis and graph plot: GraphPad Prism-9.5.0

For manuscripts utilizing custom algorithms or software that are central to the research but not yet described in published literature, software must be made available to editors and reviewers. We strongly encourage code deposition in a community repository (e.g. GitHub). See the Nature Portfolio guidelines for submitting code & software for further information.

Data

Policy information about availability of data

All manuscripts must include a data availability statement. This statement should provide the following information, where applicable:

- Accession codes, unique identifiers, or web links for publicly available datasets
- A description of any restrictions on data availability
- For clinical datasets or third party data, please ensure that the statement adheres to our policy

All the data are included in the manuscript and any additional information required to reanalyse the data reported in this paper is available from the corresponding author upon reasonable request. The cryo-EM maps and structures have been deposited in the PDB and EMDB with accession numbers 8IY9 and EMD-35817 for niacin-GPR109A-Go, 8JER and EMD-36193 for acipimox-GPR109A-Go, 8IYW and EMD-35831 for GSK256073-GPR109A-Go, 8IYH and EMD-35822 for MK6892-GPR109A-Go and EMD-36280, PDB ID: 8JHN for MMF-GPR109A-Go complex. The accession codes of other PDB coordinate files referenced in this study are 8HPTI for C5a-pep bound mouse C5aR1 and 7ZL9 for inactive crystal structure of GPR109A . Source data are provided with this paper.

Research involving human participants, their data, or biological material

Policy information about studies with human participants or human data. See also policy information about sex, gender (identity/presentation), and sexual orientation and race, ethnicity and racism.

Reporting on sex and gender	No human research participants were included in this study.
Reporting on race, ethnicity, or other socially relevant groupings	No human research participants were included in this study.
Population characteristics	No human research participants were included in this study.
Recruitment	No human research participants were included in this study.
Ethics oversight	No human research participants were included in this study.

Note that full information on the approval of the study protocol must also be provided in the manuscript.

Field-specific reporting

Please select the one below that is the best fit for your research. If you are not sure, read the appropriate sections before making your selection.

☒ Life sciences ☐ Behavioural & social sciences ☐ Ecological, evolutionary & environmental sciences

For a reference copy of the document with all sections, see nature.com/documents/nr-reporting-summary-flat.pdf

Life sciences study design

All studies must disclose on these points even when the disclosure is negative.

Sample size	Sample size was not predetermined. Sample size for cryo-EM structure determination was determined by the amount of time available on the electron microscope.
Data exclusions	No data was excluded from the analysis. Particles having poor signal to noise, damaged, or exhibiting a partial complex were excluded from the data set during data processing. Including poor or junk particles would have detrimental effects on the overall resolution of the final map.
Replication	All reported results are replicable. For each experiments, we performed multiple biological replicates. Details of the number of biological replicates are mentioned in the corresponding figure legends. The determination of the structures does not require replication.
Randomization	Particle projections were randomly partitioned for resolution and quality assessment.
Blinding	No blinding was attempted or required since blinding is not necessary for structure determination and blinding was not necessary or possible for cell based or functional assays because in most cases data was collected and analyzed by individual investigator.

Reporting for specific materials, systems and methods

We require information from authors about some types of materials, experimental systems and methods used in many studies. Here, indicate whether each material, system or method listed is relevant to your study. If you are not sure if a list item applies to your research, read the appropriate section before selecting a response.

Materials & experimental systems

n/a	Involved in the study	
☐	☒	Antibodies
☐	☒	Eukaryotic cell lines
☒	☐	Palaeontology and archaeology
☒	☐	Animals and other organisms
☒	☐	Clinical data
☒	☐	Dual use research of concern
☒	☐	Plants

Methods

n/a	Involved in the study	
☒	☐	ChIP-seq
☒	☐	Flow cytometry
☒	☐	MRI-based neuroimaging

Antibodies

Antibodies used

Monoclonal Anti-FLAG® M2-Peroxidase (HRP) antibody, Sigma (Cat no. A8592) . https://www.sigmaaldrich.com/IN/en/product/sigma/a8592

ScFv16: In house purified synthetic antibody fragment used for the stabilization of protein complex. Its development has been described in https://doi.org/10.1038/s41467-018-06002-w

Validation

Monoclonal Anti-FLAG® M2-Peroxidase (HRP) antibody is commercially available and validated by the manufacturer for use .

Eukaryotic cell lines

Policy information about cell lines and Sex and Gender in Research

Cell line source(s)

Insects cell lines (Sf9) were purchased from expression system (cat no. 94-001F). HEK-293 cells were obtained from ATCC (cat. no. CRL-3216)

Authentication

None of the cell lines used were authenticated.

Mycoplasma contamination

Cell lines were not tested for mycoplasma contamination.

Commonly misidentified lines
(See ICLAC register)

No commonly misidentified cell lines were used.

Plants

Seed stocks

N/A

Novel plant genotypes

N/A

Authentication

N/A
